# Second Annual Report of the Chicago Charitable Eye and Ear Infirmary

**Published:** 1860-07

**Authors:** 


### Second Annual Report of the Chicago Charitable Eye and Ear Infirmary. May, 1860.

In accordance with the Constitution and By-Laws of the Association, the Surgeons respectfully report:

That during the year ending May 1, 1860, *one hundred and seventy-seven* patients have been under treatment; namely, *one hundred and fifty-five* with diseases of the eye, and *twenty-two* with those of the ear; making an aggregate of *two hundred and ninety-two* that have been treated since the opening of the Infirmary, two years since.

The following is a classified list of the diseases which have been treated during the past year:

### DISEASES OF THE EYE.

| | | | |
|---|---|---|---|
| Wounds and Injuries | 11 | Occlusion of Pupil | 4 |
| Foreign Particles on Cornea | 3 | Trichiasis | 4 |
| Conjunctivitis, simple | 4 | Inflammation of Lids | 11 |
| " catarrhal | 9 | Cataract | 8 |
| " granular | 46 | Paralysis of Muscles | 1 |
| " diptheritic | 1 | Atrophy of Eye Ball | 1 |
| " neonatorum | 4 | Muscæ | 2 |
| " scrofulous | 5 | Conical Cornea | 1 |
| Ulcer of Cornea | 8 | Hydrophthalmia | 1 |
| Opacity of Cornea | 8 | Night Blindness | 1 |
| Staphyloma of Cornea | 3 | Xerophthalmia | 2 |
| Amaurosis | 8 | Tumor of Eye Ball | 1 |
| Cystic Fumors of Lids | 1 | Abscess ef Orbit | 1 |
| Iritis | 4 | Pterygium | 1 |
| Total | | | 155 |

DISEASES OF THE EAR.

| | | | |
|---|---|---|---|
| Ottorhœa | 6 | Perforation of Membrana Tympani | 4 |
| Tinnitus | 4 | Foreign bodies in External Meatus | 3 |
| Inflammation of External Meatus | 2 | Not classified | 4 |
| Total | | | 22 |

Of these, *one hundred and sixty* were natives of foreign countries, and *seventeen* of the United States.

It is the special object of Charitable Eye Infirmaries to furnish the afflicted poor with suitable medical advice and aid, before loss or diminution of vision has become imminent. This work is essentially preventive and remedial, and is designed to relieve society beforehand of burdens, which, if the patient finds no seasonable relief, will fall heavily upon private friends or the public at large. No charity can be more wise, economical and worthy. Few forms of benevolence can accomplish so large a good at so small a cost.

The Dispensary of the Infirmary, at No. 60 North Clark Street, is open daily, from 11½ to 1 o'clock, for the gratuitous treatment of the poor, afflicted with diseases of the Eye or Ear.

The attending Surgeons are Edward L. Holmes, M. D., and Edwin Powell, M. D.

